# Computational Workflow to Design Novel Vaccine Candidates and Small-Molecule Therapeutics for Schistosomiasis

**DOI:** 10.3390/pathogens13100850

**Published:** 2024-09-30

**Authors:** Emmanuel Oluwadare Balogun, Gideon Ibrahim Joseph, Samuel Charles Olabode, Naziru Abdulkadir Dayaso, Ammar Usman Danazumi, Rachael Bashford-Rogers, James H. Mckerrow, Ghulam Jeelani, Conor R. Caffrey

**Affiliations:** 1 Department of Biochemistry, Ahmadu Bello University, Zaria 810001, Kaduna, Nigeria; samuelpsalmbodex3@gmail.com (S.C.O.);; 2 Africa Center of Excellence for Neglected Tropical Diseases and Forensic Biotechnology (ACENTDFB), Ahmadu Bello University, Zaria 810001, Kaduna, Nigeria; 3 Center for Discovery and Innovation in Parasitic Diseases, Skaggs School of Pharmacy and Pharmaceutical Sciences, University of California San Diego, 9500 Gilman Drive, MC0657, La Jolla, CA 92093, USA; 4 Department of Biomedical Chemistry, Graduate School of Medicine, The University of Tokyo, Tokyo 113-0033, Japan; 5 Department of Biochemistry, Federal University of Technology, Minna PMB 65, Niger, Nigeria; josephgideonbc@gmail.com; 6 Africa Centre of Excellence for Mycotoxin and Food Safety, Federal University of Technology, Minna PMB 65, Niger, Nigeria; 7 Department of Biochemistry, University of Oxford, Oxford OX1 3QU, UK

**Keywords:** *Schistosoma*, drug and vaccine, immunoinformatics, cathepsin proteases

## Abstract

Human schistosomiasis, caused by the *Schistosoma* trematode, is a neglected parasitic disease affecting over 250 million people worldwide. There is no vaccine, and the single available drug is threatened by drug resistance. This study presents a computational approach to designing multiepitope vaccines (MEVs) targeting the cercarial (CMEV) and schistosomular (SMEV) stages of schistosomes, and identifies potential schistosomicidal compounds from the Medicine for Malaria Ventures (MMV) and SuperNatural Database (SND) libraries. The designed vaccines (CMEV and SMEV) are engineered to provoke robust immune responses by incorporating a blend of T- and B-cell epitopes. Structural and immunoinformatics evaluations predicted robust interactions of CMEV and SMEV with key immune receptors and prolonged immune responses. In addition, molecular docking identified several compounds from the MMV and SND libraries with strong binding affinities to vital *Schistosoma* cathepsin proteases, indicating their potential as schistosomicidal agents. Our findings contribute to the potential development of effective vaccines and drugs against schistosomiasis.

## 1. Introduction

Human schistosomiasis is a neglected parasitic disease that is caused by species of the trematode blood fluke, *Schistosoma*. The disease is of global public health importance, with over 229 million people infected in 2015 [1], ranking as the third most prevalent parasitic illness and the second most important in terms of socioeconomic impact [2]. Infection is also associated with immunosuppression and carcinogenic effects and is estimated to account for an annual global death range of 11,700–280,000 [3,4].

Medically important *Schistosoma* species include *Schistosoma japonicum*, *Schistosoma haematobium*, and *Schistosoma mansoni*. *S. japonicum* causes intestinal schistosomiasis in China, Indonesia, and the Philippines. *S. haematobium*, which is common in Africa, is transmitted by *Bulinus* snails and leads to urogenital schistosomiasis, while *S. mansoni*, also prevalent in Africa, results in intestinal and hepatic forms of schistosomiasis [5]. Schistosoma species are capable of zoonosis, with animal hosts such as wild rodents serving as reservoirs, facilitating the transmission of these parasites to humans, especially in regions where ecological barriers have broken down [6].

The intricate life cycle of the schistosome involves two hosts: the snail intermediate host and the definitive human host. Eggs are released in urine and/or feces. In water, these hatch to release free-swimming miracidia. These penetrate the snail and proliferate as sporocysts via asexual reproduction and terminally differentiate into cercariae, which are released into the water to infect humans and other mammals. Upon penetration of the skin, cercariae transform into schistosomula, which enter the venous blood circulation via the lungs to grow as males or females, mate, and lay eggs [7].

Praziquantel (PZQ) is the sole anti-schistosomal drug on the market, having been developed in the 1970s [8]. It is considered effective for morbidity control, with variable cure rates between 60 and 95%, depending on factors such as the *Schistosoma* species, infection intensity, and regional differences in treatment outcomes [9,10]. However, PZQ has several pharmaceutical and pharmacological drawbacks [11,12], among which is its lack of efficacy against juvenile worms 2–4 weeks post-infection [13], reduced oral bioavailability, and rapid metabolic clearance by the host [14,15]. Apart from the paucity of drug options, there is no vaccine, underscoring the need to develop new therapeutic modalities to control schistosomiasis [2,16]. It is considered that vaccination, either alone or in conjunction with chemotherapy, offers the most promising approach for the long-term management and eventual eradication of schistosomiasis [17].

A multiepitope vaccine is a chimera of multiple epitopes from different antigens and is capable of stimulating linear B lymphocytes (LBLs), cytotoxic T lymphocytes (CTLs), and helper T lymphocytes (HTLs). The utilization of immunoinformatics and structural biology tools in vaccine design represents a significant advancement over traditional methods, offering a faster, cost-effective, and potentially safer route to vaccine development. The identification of multiple antigenic, non-allergenic, and non-toxic epitopes ensures the vaccine’s potential efficacy and safety, critical for its application in diverse populations [2]. This approach has led to the design and development of chimeric vaccines against various diseases, including trypanosomiasis, monkeypox, brucellosis, cholera, toxoplasmosis, dengue fever, onchocerciasis and other filarial diseases, COVID-1, and cancer [2,18,19,20,21,22,23,24]. Of note, EMD640744, a chimeric vaccine designed against solid malignancies, has shown great promise and has successfully completed a phase 1 clinical trial [25].

Beyond vaccine development, the hemoglobin degradation pathway in the adult schistosome is an attractive target for drug discovery as it plays a critical role in the parasite’s survival in the host [26,27,28]. Specifically, *Schistosoma* cysteine (cathepsins B1, C, and L) and aspartic proteases (cathepsin D) contribute to the degradation of host hemoglobin as a nutrient source for growth and egg production [29,30,31,32]. Inhibitors specific to cathepsin B1.1 are well advanced and RNA interference of *S. mansoni* cathepsins B1.1, C, and D decreases the parasite’s viability [32,33,34,35,36]. Last, gene expression analysis has equally shown that these protease transcripts are upregulated in cercariae and schistosomula [25,26].

**Figure 1:**
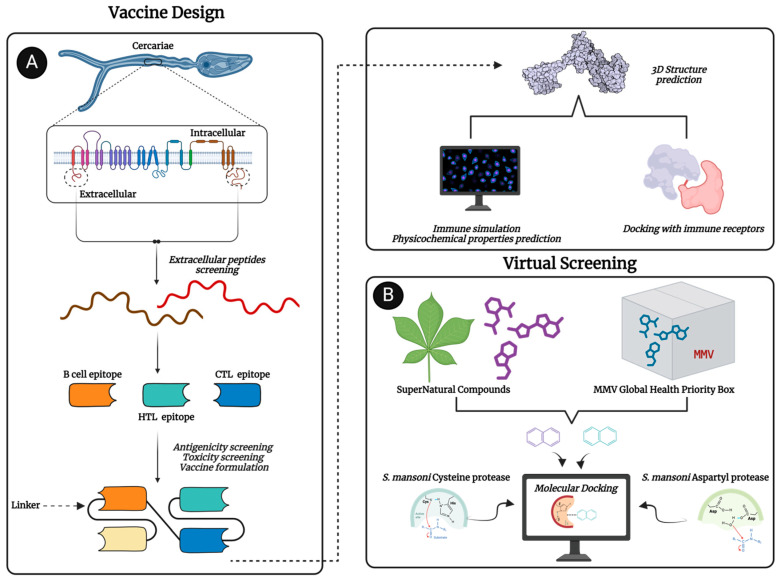
Flowchart of computational (**A**) vaccine design against *Schistosoma mansoni* membrane proteins and (**B**) Virtual screening against *Schistosoma mansoni* cathepsins. Created with BioRender.com.

Accordingly, we first conceived and computationally designed multiepitope vaccines targeting two important developmental stages of schistosomes, cercariae and schistosomula. The vaccine constructs (CMEV and SMEV) were designed to provoke robust immune responses by incorporating a blend of T-cell and B-cell epitopes (Figure 1). We then employed virtual screening to identify potential anti-schistosomal compounds from Medicine for Malaria Venture’s (MMV) Global Health Priority (GHP) Box [37] and the SuperNatural database (SND) (Figure 1). Our screening identified seven compounds that matched or exceeded the predicted binding energies of the standard protease inhibitors E64 and pepstatin against *Schistosoma* cysteine and aspartic proteases, highlighting their potential to inhibit the parasitic cathepsins.

## 2. Materials and Methods

### 2.1. Selection of Protein Targets

Protein-encoding genes reported to be upregulated in both cercariae and schistosomula of *S. mansoni* were identified [37], and the corresponding protein sequences were downloaded from the UniProt database (Supplementary data sheet S1). DeepTMHMM was used to predict proteins with transmembrane helices [38]. DeepTMHMM is a deep neural network-based approach that predicts protein topology using the target protein’s amino acid sequence by a pre-trained protein language model [38]. Proteins harboring transmembrane helices were further submitted to DeepLoc [39], which uses a UniProt-trained neural networks algorithm to predict subcellular localization [39]. Proteins predicted to be localized in the plasma membrane were selected and extracellular peptides from those proteins that were ≥9 amino acid residues long were selected for further analysis. Finally, the selected extracellular peptides were evaluated for orthology with human proteins using NCBI BLASTP [40]. Protein sequences with similarities to human orthologs of ≥35% and an E-value >0.0001 were excluded.

### 2.2. Prediction of Cytotoxic T Lymphocyte (CTL) and Helper T Lymphocyte (HTL) Epitopes

The selected extracellular helices were submitted to NetMHCIIPan-4.0 and NetMHCPan-4.1 [41,42,43] to predict binders of major-histocompatibility complex I and II (MHCI and MHCII), respectively. The NetMHCpan-4.1 server predicts MHCI binders based on a neural network that is pretrained using data from binding affinity (BA) and eluted ligand mass spectrometry (EL) [41,42]. The NetMHCIIpan-4.0 server predicts peptide binding to an MHC II molecule of a known sequence using artificial neural networks (ANNs). It is trained on an extensive dataset of over 500,000 measurements of BA and EL, covering the three human MHC class II isotypes, HLA-DR, HLA-DQ, HLA-DP, as well as mouse major histocompatibility complex (H-2) molecules [43].

### 2.3. B-Cell Epitope Prediction

The extracellular helices were also submitted to the BepiPred-3.0 server [44] to predict linear B-cell epitopes. BepiPred-3.0 is a sequence-based epitope prediction tool that contains embedded protein language models (LMs), which significantly increase the prediction accuracy (relative to BepiPred-2.0 [45]) for both linear and conformational epitopes based on several independent test sets.

### 2.4. Selection of Overlapping Epitopes

CTL/HTLs and B-cell epitopes were aligned with MUL-TALIN [46], and overlapping epitopes were selected using the server’s default parameters. They have 9 to 71 amino acids.

### 2.5. Interferon Gamma (IFN-ɣ)-Inducing Epitopes

Given that the production of IFN-ɣ has been associated with resistance to schistosome infection [47,48], the final set of HTL epitopes was submitted to the IFN-ɣ-epitope server [49] to assess their ability to induce interferon-gamma production.

### 2.6. Antigenicity, Allergenicity, and Toxicity Analysis

The selected CTL, HTL, and B-cell epitopes were submitted to VaxiJen 2.0 [50] to predict antigenicity using a threshold of ≥0.5 for parasites. This threshold value has been pre-established to be sufficient for the prediction of antigenicity in parasite proteins [50]. The epitopes were additionally submitted to AllerTOP [51] for allergenicity prediction. In this server, allergen prediction uses amino acid descriptors that consider the size, quantity, and α-helix- and β-strand-forming propensities and hydrophobicity of residues. The protein allergens and non-allergens were categorized using a machine learning technique utilizing k nearest neighbors [51]. Finally, the toxicity of the peptides was predicted using the Toxinpred-3.0 server using the server’s default threshold of 0.38 [52]. Toxic peptides were discarded and non-toxic peptides were selected for subsequent analyses.

### 2.7. Construction of Vaccine Candidates

**Figure 2:**
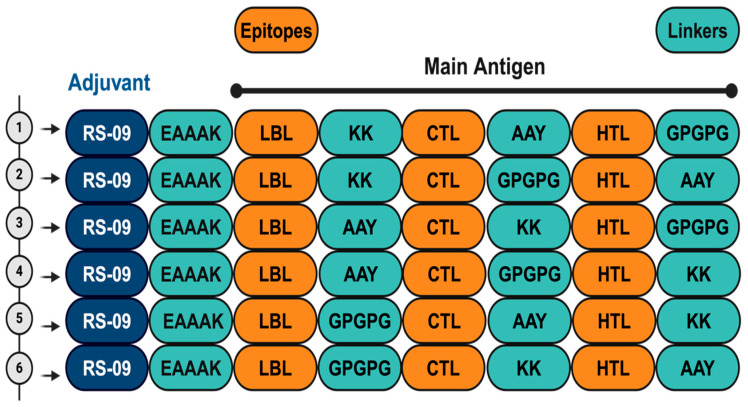
Order of construction of vaccine candidates (created with BioRender.com). This is a generic figure showing how linkers were reshuffled between epitopes to create 6 constructs for each growth stage. *Linkers* Linker sequences. *LBL* Linear B-cell epitopes. *CTL* cytotoxic T cell epitopes. *HTL* Helper T cell epitopes.

Multiepitope vaccines were designed using peptide sequences consisting of T- and B-cell epitopes that were predicted to be antigenic, non-allergenic, and non-toxic. Epitopes were classified as belonging to upregulated proteins in either cercariae or schistosomula, and separate vaccine constructs were designed for each developmental stage. Each construct starts with the TLR4 agonist, APPHALS (RS-09), linked with an EAAAK linker.RS-09 was added to activate TLR4 by imitating the receptor’s interaction with LPS, and incorporating it into the construct allows for a ready-to-use formulation without the need for an additional adjuvant. This is followed by linear B-cell epitopes, CTL epitopes, and finally HTL epitopes. AAY, GPGPG, and KK linkers, which are rigid, flexible, and cleavable linkers, were used to connect different classes of epitopes. The initial assignment of the position of the linkers was random and then later reshuffled among the epitope classes to obtain the best Ramachandran statistics (Figure 2). Subsequently, each multiepitope vaccine construct was submitted to the VaxiJen 2.0, AllerTOP server, and Toxinpred-3.0 servers [50,51,52] to predict antigenicity, allergenicity, and Toxicity of the designed antigens. Finally, the vaccine constructs were aligned against the human proteome using the NCBI P-BLAST to exclude constructs with >35% homology to human proteins.

### 2.8. Vaccine 3D Structure Prediction and Physicochemical Properties Evaluation

The 3D structures of the designed putative antigens were predicted using RoseTTAFold from the Robetta webserver [53]. The predicted structures were further evaluated on PROCHECK from the SAVES server [54]. In addition, the physicochemical properties of all constructs were predicted using Expasy Protparam [55]. Two constructs, one each for cercariae and schistosomula, were selected based on having the least Ramachandran outliers (Tables S1 and S2).

### 2.9. Molecular Docking of Construct with Immune Receptors

Given that toll-like receptors 2, 3, and 4 (TLR2, TLR3, and TLR4, respectively) are essential to stimulation of immune response against schistosomes, the predicted 3D structures of the constructs were docked against human TLR2, TLR3, and TLR4 using the ClusPro protein–protein docking server [56]. The protein databank accession codes 6NIG, 7C76, and 2Z63 were used to retrieve TLR2, TLR3, and TLR4, respectively, and co-crystallized substrates and ligands were removed prior to docking. The server’s default parameters were used for the docking. The center of the lowest energy cluster was selected for each construct.

### 2.10. Immune Response Simulation

The two selected constructs (one for each stage) were submitted to the C-IMMSIM server [57] to simulate immune response against the putative antigens. The server utilizes a machine learning-based algorithm with position-specific scoring matrices to model immune interactions. The simulation spanned 366 days, equivalent to 1098 steps, with each step taking 8 h. Virtual injections were administered at steps 1, 84, and 168 to simulate three doses of the antigens.

### 2.11. Selection of Cathepsin Drug Targets and Multiple Sequence Alignment

The following FASTA sequences were retrieved from UniProtKB (https://www.uniprot.org/ accessed on 2 December 2023): *S. mansoni* cathepsins B1.1 (Q8MNY2), C (Q26563), L (Q26534) and D (P9180). *S. haematobium* cathepsins B (A0A095A1C7), D (A0A6A5DMA6) and L (A0A095A5Y5), and a dipeptidyl-peptidase (A0A6A5DBI8). *B. taurus* cathepsins B (P07688), C (A0AAA9RYR4), D (P80209) and L (A0A3S5ZPJ8). *R. norvegicus* cathepsins B (P00787), C (A0A8I6A0Q1), D (A6HY44) and L (A0A8L2QDP6). *H. sapiens* cathepsins B (P07858), C (A0A7I2YQT5), D (P07339) and L (A0A7I2YQA2). *Plasmodium falciparum* falcipain2 (3PNR), dipeptidyl-peptidase I (A0A0L1IF06) and plasmepsin II (P46925). Multiple sequence alignments (MSAs) were performed using the ClustalO server (https://www.ebi.ac.uk/jdispatcher/msa/clustalo, accessed on 15 May 2024) to identify conserved motifs/residues and catalytic residues.

### 2.12. Molecular Modeling and Docking

The SWISS-MODEL server [58] was used to model *S. mansoni* cathepsins B1.1 (Q8MNY2), C (Q26563), L (Q26534), and D (P9180), utilizing the PDB accession codes structures 4i04.1.A, 6CZS, 3F75, and 5UX4, respectively, as templates. The generated models were prepared for docking using the UCSF chimera version 1.16 [59] to generate the pdbqt format.

To identify potential inhibitors of the above-listed proteases, 227 compounds from the Medicine for Malaria Venture’s (MMV) Global Health Priority (GHP) Box [37] and 442 natural compounds from the SuperNatural database (SND; http://bioinformatics.charite.de/supernatural, accessed on 2 December 2023) [60] were used. The MMV compounds were previously utilized in zoonotic and neglected diseases, drug-resistant malaria, and vector control research, while the compounds from the SND library were selected based on their ability to be orally administered and gastro-intestinally absorbed [60]. The common cysteine and aspartic proteases inhibitors, E64 (PubChem 123985) and pepstatin A (PubChem 5478883), respectively, were minimized using the uff forcefield [61] and the steepest descent optimization algorithm [62], and then converted to pdbqt for docking. The vina wizard contained in the PyRx autodock suite was used as the docking algorithm [63]. The grid was maximized for each of the cathepsins, and a blind docking strategy was adopted to obtain binding energies and docking poses with reliable predictions, while all other parameters were left as default. Compounds with binding affinities above that of the respective inhibitor, without violation of Lipinski’s rule-of-five parameters [64], and not interacting with more than two cathepsin targets from the virtual screening, were selected as hits.

## 3. Results

### 3.1. Selection of Extracellular Helices

A total of 72 and 105 proteins that are upregulated and highly expressed in cercariae and schistosomula, respectively, were retrieved from the UniProt server and analyzed using DeepTMHMM to predict proteins with transmembrane helices. Of these, subcellular localization analysis revealed 18 and 46 predicted plasma membrane proteins in cercariae and schistosomula, respectively, that lacked orthology with human proteins. From these, 40 and 73 extracellular peptides in cercariae and schistosomula, respectively, were identified for further downstream analyses (Figure 1).

### 3.2. Prediction of CTL, HTL, and B-Cell Epitopes

Of the 40 extracellular peptides from cercariae, NetMHCpan and NetMHCIIpan predicted a total of 79 and 10 CTL and HTL epitopes, respectively. Prediction of linear B-cell epitopes using Bepipred-3.0 gave a total of 12 peptides. Of the 73 extracellular peptides in schistosomula, NetMHCpan and NetMHCIIpan predicted a total of 183 and 27 CTL and HTL epitopes, respectively. Bepipred-3.0 gave a total of 19 linear B-cell epitopes.

### 3.3. Antigenicity, Allergenicity, and Toxicity Analysis

The predicted CTL, HTL, and B-cell epitopes were subjected to antigenicity, allergenicity, and toxicity analysis, as described above. For cercariae, 9 LBL, 19 CTL, and 5 HTL (1 strong and 4 weak binders) epitopes were identified, respectively (Table S3). For schistosomula 10 LBL, 17 CTL, and 2 HTL (3 strong binders and 6 weak binders) epitopes were identified, respectively (Table S3). Weak binders were included for their abilities to induce the production of IFN-ɣ.

### 3.4. Vaccine Candidates

**Figure 3:**
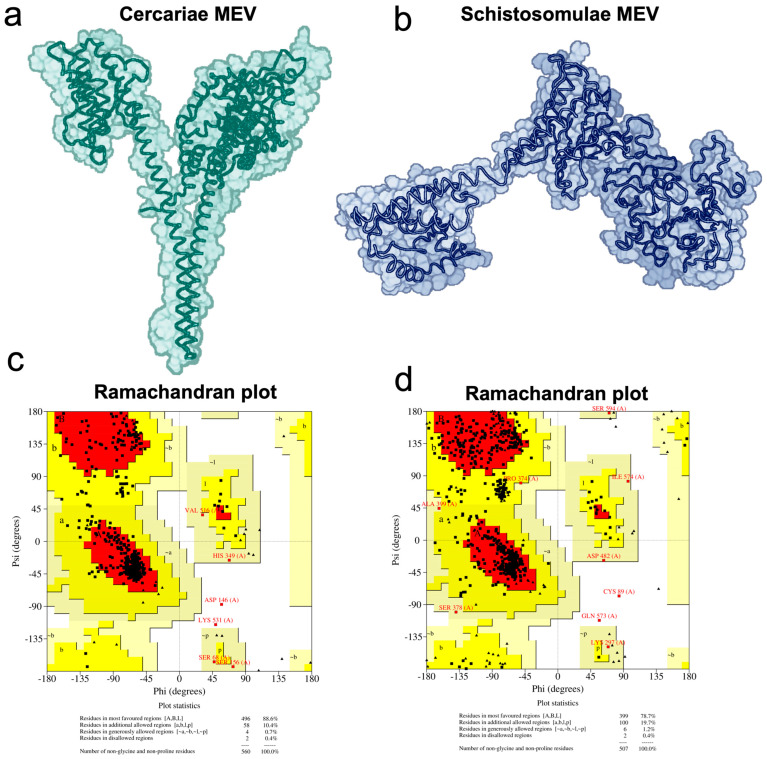
The predicted 3D structures of selected vaccine constructs designed to target *Schistosoma mansoni* cercariae (**a**) and schistosomula (**b**), and (**c**,**d**), the respective Ramachandran plots including the statistics for those residues in favored, allowed, and disallowed regions.

The vaccine constructs were designed from the above-predicted epitopes using rigid, flexible, and cleavable linkers. For all constructs, the TLR-4 agonist RS-09 was used as adjuvant and linked with the first epitope using EAAAK as the linker. Different constructs were designed to target either cercariae or schistosomula by joining a LBL with a CTL and HTL using different combination of linkers, as shown in Figure 2. Six putative antigens were constructed for both cercariae and schistosomula, and their 3D structures were predicted using RoseTTAFold from the Robetta webserver (Figure 2 and Figure S1). The predicted structures were evaluated on the SAVES server, and the best structures were selected based on their Ramachandran outlier values. Accordingly, construct 1 (CMEV) and construct 4 (SMEV) were selected as the best candidates and were subjected to subsequent analyses (Figure 3).

### 3.5. Interaction of the Vaccine Candidates with Key Immune Receptors

**Figure 4:**
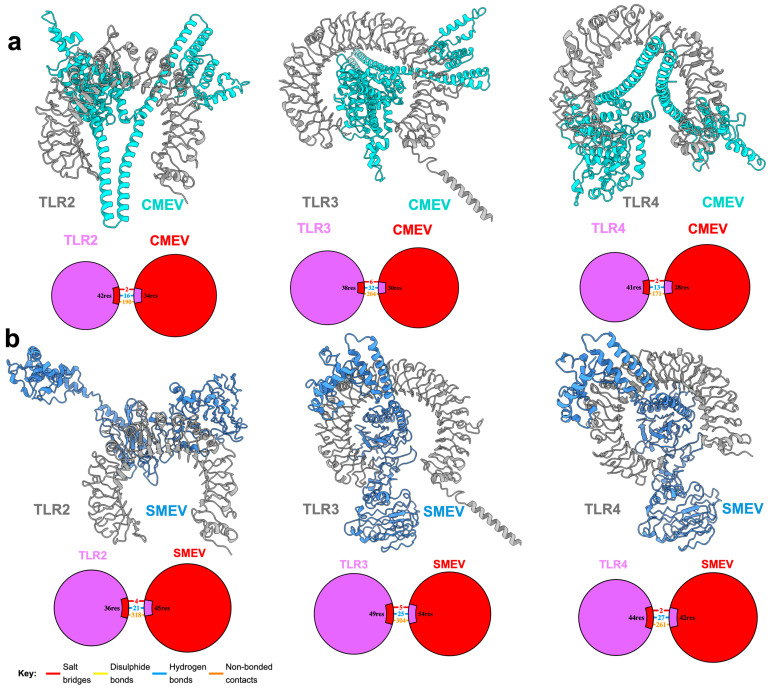
Predicted binding modes and summary of the interaction of CMEV (**a**) and SMEV (**b**) to human TLR2, TLR3, and TLR3.

Due to the role of human TLR2, TLR3, and TLR4 in the stimulation of response against schistosomes, such as cytokine production and stimulation of dendritic cell (DC) maturation and activation [65], CMEV and SMEV were probed against these receptors for possible interactions. The putative antigens were predicted to intimately interact with all three immune receptors (Figure 4). CMEV demonstrated a robust interaction with TLR3, forming 32 hydrogen bonds, six salt bridges and 204 additional non-bonded interactions, including van der Waals and electrostatic interactions. In comparison, TLR2 and TLR4 formed 16 and 13 hydrogen bonds with CMEV, respectively. Additionally, they each formed two salt bridges with the antigen, and 190 and 171 non-bonded interactions were recorded for TLR2 and TLR4, respectively (Figure 4a). In contrast, SMEV exhibited comparable interactions with the three immune receptors, forming 21, 25, and 27 hydrogen bonds with TLR2, TLR3, and TLR4, respectively. In addition to forming hydrogen bonds, SMEV established four salt bridges and 318 non-bonded interactions with TLR2, five salt bridges and 304 non-bonded interactions with TLR3, and two salt bridges and 261 non-bonded interactions with TLR4 (Figure 4b). Overall, both CMEV and SMEV are predicted to interact extensively with each of the TLRs, indicating their potential to stimulate a strong immune response.

### 3.6. Immune Response Simulation

**Figure 5:**
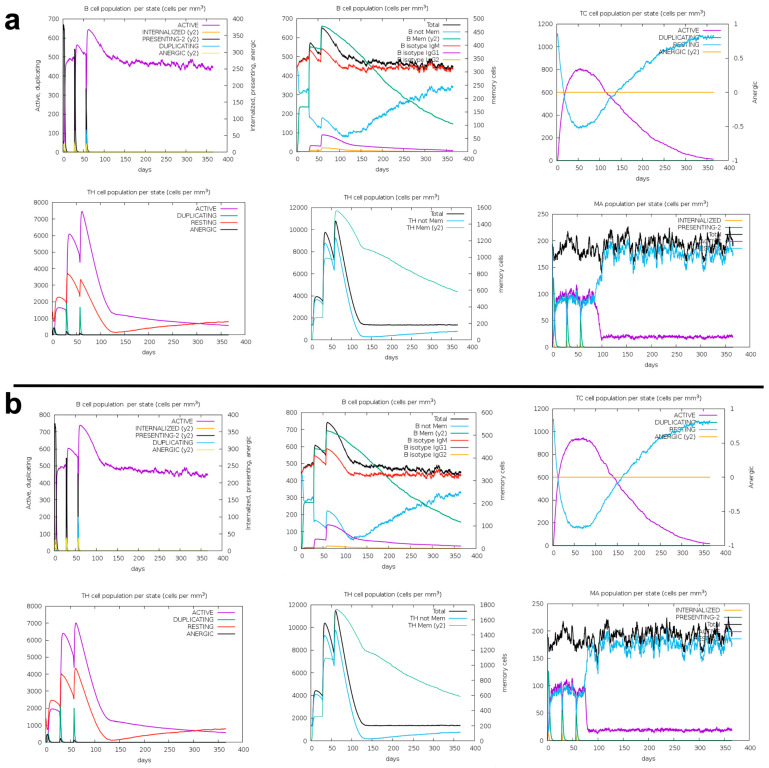
Immune response simulation against CMEV (**a**) and SMEV (**b**). Three doses of each putative antigen were administered, and the simulation lasted for 1 year.

The immune response to the designed putative antigens was extensively simulated using the C-IMMSIM server. This server utilizes advanced position-specific scoring matrices to accurately model immune interactions. During the simulation, three doses of CMEV or SMEV were administered at specific intervals. This regimen resulted in a prolonged and robust activation of both B-cell and cytotoxic T-cell populations (Figure 5a,b). Furthermore, helper T cell populations were also significantly activated by both antigens (Figure 5a,b). However, it is noteworthy that a decline in these populations was observed after 150 days, indicating a temporal aspect to this class of immune response (Figure 5a,b). Nonetheless, the activities of macrophages were markedly stimulated and sustained throughout the entire simulation period. Taken together, these findings strongly suggest that both CMEV and SMEV will elicit the desired immune response, demonstrating their potential efficacy as vaccine candidates.

### 3.7. Identification of Potential Inhibitors of Schistosoma Cathepsins

As described previously, gene expression analysis revealed the upregulation of *Schistosoma mansoni* cathepsin B (all isotypes), D, and L, while RNA interference confirmed the essential role of cathepsin B1 and D in the parasite’s survival [31,32,37,66]. Consequently, we virtually screened 227 and 442 compounds from the MMV GHP box and SND library, respectively, against *S. mansoni cathepsins B1.1 (Q8MNY2)*, *C (Q26563)*, *L (Q26534)*, and *D (P9180)* cathepsins (Supplementary data sheet S2).

To compare the potency of the compounds from these two libraries, the cysteine and aspartic protease inhibitors, E-64 and pepstatin A, respectively, were used as reference standards. To validate our docking protocol, the standard inhibitors E64 and pepstatin were docked against human cathepsins B, D, and L. Their predicted binding poses were compared with the respective crystal structures of these cathepsins bound to E-64 or pepstatin. The docking recapitulated the crystallographic observations, demonstrating the reliability of our protocol (Figure S2). Consequently, compounds performing better than the standard inhibitors (in terms of binding energy) were selected as hit compounds for each cathepsin, resulting in a total of 27 and 88 compounds from MMV and SND libraries, respectively (Supplementary Datasheet S2).

Accordingly, MMV1794209 from the MMV library and UNPD221842 from the SND library outperformed E-64 against cathepsin B1.1 (SmCB), whereas only UNPD28979 from the SND library matched the score of E-64 against cathepsin C (SmCC) (Table 1, Supplementary data sheet S1). Against cathepsin D (SmCD), MMV979319 from the MMV library matched the score of pepstatin, whereas UNPD125303 from the SND library outperformed pepstatin. Against cathepsin L (SmCL), E64 was outperformed by MMV1577465 and UNPD73743 from the respective MMV and SND libraries (Table 1, Supplementary data sheet S1).

**Figure 6:**
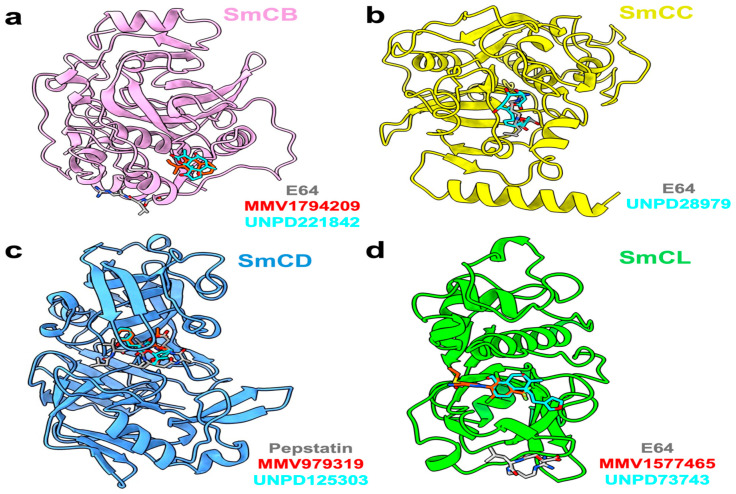
Binding modes of selected compounds from MMV and SND libraries against SmCB (**a**), SmCC (**b**), SmCD (**c**) and SmCL (**d**).

**Figure 7:**
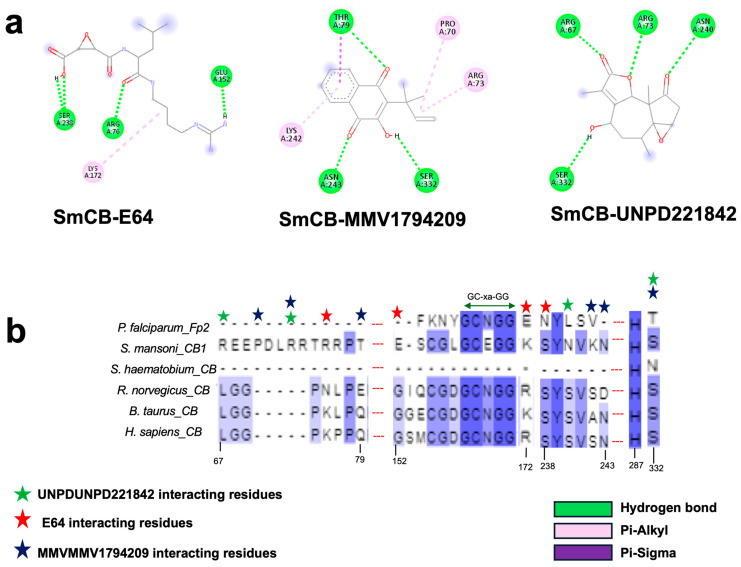
Two-dimensional interaction of SmCB with hit compounds. (**a**) Interaction of E64, MMV1794209, and UNPD221842 with SmCB. (**b**) Multiple sequence alignment of SmCB protein sequence against selected species.

Examining the ligand binding modes, MMV1794209 and UNPD221842 occupied the same pocket in SmCB, which is distinct from the binding pocket of the standard inhibitor E-64 (Figure 6a). MMV1794209 interacts with SmCB through three hydrogen bonds, three π-alkyl interactions, and an additional Pi–sigma interaction (Figure 7a). Similarly, UNPD221842 engages with SmCB via four hydrogen bonds, unlike E-64, which forms three hydrogen bonds and a π–alkyl interaction (Figure 7a). This interaction pattern elucidates why MMV1794209 and UNPD221842 outperformed E-64. Importantly, none of these compounds interact with a catalytic cysteine or histidine, indicating they are unlikely to interfere with the enzyme’s catalytic activity. However, multiple sequence alignment reveals that S332, which interacts with both MMV1794209 and UNPD221842, and N243, which interacts only with MMV1794209, are conserved in the host cathepsin B (Figure 7b). Therefore, a structure-activity relationship (SAR) study may be necessary to ensure these compounds selectively target the parasite protease without affecting the host protease.

UNPD28979, the only candidate that matched E-64 for binding to SmCC, occupied an identical binding pocket as E-64 (Table 1), with the two ligands being nearly superimposable (Figure 6b). Unsurprisingly, these ligands are coordinated by mostly identical residues in SmCC and exhibit similar interaction types (Figure S3a). Notably, binding residues such as V366, E394, and N397 are common to both ligands and are conserved in the mammalian host (Figure S3b). Nevertheless, catalytic residues are not involved in both interactions.

For SmCD, although MMV979319 only engaged the target through hydrophobic and electrostatic interactions without forming any hydrogen bonds, it surprisingly matched pepstatin in binding energy (Figure 6c). Pepstatin formed four hydrogen bonds with SmCD, as well as other non-polar interactions (Figure S4a). Similarly, UNPD125303 engaged SmCD with four hydrogen bonds and two other non-polar contacts involving Y128 and D83, which were also maintained in the interaction with MMV979319 (Figure S4a). This observation suggests that UNPD125303 is a better binder of SmCD than either pepstatin or MMV979319. Of note, all of the SmCD residues interacting with pepstatin, MMV979319, and UNPD125303 are conserved in human cathepsin D, with the exception of T130 (Figure S4b). Moreover, the DTG catalytic motif of cathepsin D participates in the interaction with all compounds (Figure S4), suggesting the possible modulation of the enzyme’s activity.

Finally, for SmCL, MMV1577465 and UNPD73743 are positioned closely in a wide binding pocket that is distinct from the binding site of E-64 (Figure 6d). SmCL forms four hydrogen bonds with E-64 through H248, two hydrogen bonds with MMV1577465 through H265 and Q244, and three hydrogen bonds with UNPD73743 via Q123, M125, and W288 (Figure S5a). H265, which interacts with MMV1577465, and Q123, which interacts with UNPD73743, are both conserved in human cathepsin L (Figure S5b). Notwithstanding this, active sites residues are not involved in these contacts. In general, compounds from the SND library established more robust interactions with the cathepsin targets than those from the MMV library (Table 1, Figure 6 and Figure S2–S4). However, residues coordinating the interaction of most compounds, and the established inhibitors, are conserved in the human orthologs, suggesting the potential need for structure-activity relationship-guided optimization.

## 4. Discussion

We have presented the computational design of multiepitope vaccines targeting the cercariae (CMEV) and schistosomula (SMEV) stages of schistosome parasites. Additionally, we virtually screened a library of natural compounds and a chemical library from Medicines for Malaria Ventures (MMV) curated to target infectious agents. The vaccines (CMEV and SMEV) were designed from a repertoire of extracellularly exposed peptides derived from *S. mansoni* transmembrane proteins. In general, surface proteins from pathogens are more prone to engage their host immune effectors, making them more likely to trigger immune response [67]. The selected peptides offered epitopes that bind to cytotoxic T lymphocytes (CTLs), helper T lymphocytes (HTLs), and B cells. This is crucial for eliciting a comprehensive immune response, including the production of neutralizing antibodies, activation of CTLs for pathogen clearance, and support from helper cells to enhance the overall immune reaction.

Epitopes were linked using EAAAK, KK, GPGPG, and AAY linkers. These linkers not only connect the epitopes, but also enhance expression yield, facilitate immune processing and presentation, and promote favorable pharmacokinetic profiles [42,66]. In addition to the linkers, the adjuvant RS-09, a TLR4 agonist, was incorporated at the N-terminal of all constructs. TLR4 agonists are emerging as potent natural adjuvants that stimulate both innate and adaptive immune responses. They enhance the production of pro-inflammatory cytokines and activate antigen-presenting cells (APCs) [2,68].

While several multiepitope vaccines (MEVs) have been designed to target schistosomes by incorporating a combination of immunogenic epitopes to trigger protective immune responses [2,16,69], our approach distinguishes itself by leveraging the parasite’s gene expression patterns to identify stage-specific antigens [70]. This allows us to design a vaccine that aligns more closely with the parasite’s developmental biology, potentially increasing its efficacy by targeting proteins that are crucial at specific stages of the parasite’s life cycle.

Beyond vaccine design, our study investigated the potential of inhibiting schistosome cathepsins, which are key proteins involved in hemoglobin catabolism within the parasite. Using virtual screening, we probed the combination of compounds from SND and MMV libraries against SmCB, SmCC, SmCD, and SmCL, and identified compounds with superior predicted binding affinity than the standard protease inhibitors E64 and pepstatin. Unlike E64 and pepstatin, which are active site inhibitors [71,72], the potential inhibitors (Table 1) identified in this study do not engage SmCB, SmCC, and SmCL active site residues. In contrast, UNPD125303 and MMV979319, like pepstatin, do act on the SmCD active site, suggesting the possible modulation of the enzyme’s activity.

Among these compounds, MMV1794209 (2-hydroxy-3-(2-methylbut-3-en-2-yl)naphthalene-1,4-dione), known as Dunniol, is a naphthoquinone found in the tuber of Brazilian herbs of the genus *Sinningia* [73,74,75,76]. It is demonstrated to possess in vitro antitumoral photoinduced effects against melanoma and prostate cancer cell lines [76] and is designated as a vector control agent in the Medicine for Malaria Venture’s Global Health Priority Box (MMV’s GHPB). Similarly, MMV1577465, or Ethiprole (5-amino-1-[2,6-dichloro-4-(trifluoromethyl)phenyl]-4-ethylsulfinylpyrazole-3-carbonitrile), is a phenylprazole, and a widely utilized agricultural insecticide as an alternative to fipronil, probably because of its lower toxicity and persistence in the environment [77,78]. It is a broad-spectrum systemic insecticide that is effective against insect pest species such as pentatomoids, fulgoroids, aphidoids, dipterans, caeliferans, and boll weevils, amongst many others [76]. Its mode of action involves inhibiting the insect’s chloride ion channel ᵞ-aminobutyric acid receptor [79,80]. Although ethiprole possesses enantioselective endocrine-disrupting effects and toxic neurobehavioral effects in animal models [77,81], its actual dietary intake limit (0.005 mg/kg bw) suggests it is unlikely to produce adverse effects in humans [81].

Finally, MMV979319 (2-[2-[(3-methyl-1-pyridin-2-ylbutyl)amino]ethyl]phenol) is designated as a small-molecule compound against drug-resistant malaria in the MMV’s GHPB. Unlike the well-studied compounds from the MMV library, those from the SND libraries have not been well investigated, and there appears to be little information available about their activities and applications. Hence, our study offers a framework for assessing the potential of these natural compounds and using the data to update the collection of anti-*Schistosoma* compounds. Future work should prioritize the expression and purification of the MEVs, along with comprehensive in vitro and in vivo validation of their efficacy and that of the potential drug candidates.

## 5. Conclusions

This study describes in silico workflows that predict multiepitope vaccines and small-molecule compounds that may prove useful in the control and elimination of schistosomiasis, an infectious disease of global public heath importance. The vaccine constructs that target the cercarial and schistosomula stages are predicted to elicit strong immune responses, as evidenced by interactions with TLR receptors. Furthermore, the immune response simulations suggest that the designed vaccines will induce prolonged activation of B cells, cytotoxic T cells, and macrophages, which is essential for combating infections.

Given the importance of the hemoglobin degradation pathway to the growth and fecundity of the parasite, our study also identified novel potential inhibitors from the MMV and SND libraries, against cathepsin proteases, which may prove useful in the control and management of the disease.

Overall, our study provides a comprehensive framework for the design of potentially effective vaccines and therapeutics against schistosomiasis. The integration of advanced computational tools with experimental validation could accelerate the fight against neglected tropical diseases, ultimately improving global health outcomes. Our future work will focus on the experimental validation of the designed vaccine constructs and the identified compounds, paving the way for clinical development and potential deployment in endemic regions such as Nigeria.

## Figures and Tables

**Table 1:** Molecular docking-predicted binding energies of selected compounds from the MMV and SND libraries against *S. mansoni* cathepsins.

Target Cathepsin	Hit ID	Hit Structure	Binding Energy (kcal/mol)
B	UNPD221842	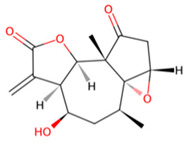	−7.6
	MMV1794209	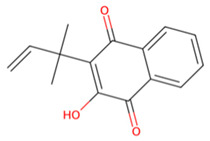	−6.7
C	UNPD28979	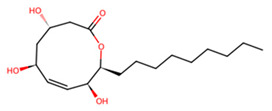	−6.7
D	UNPD125303	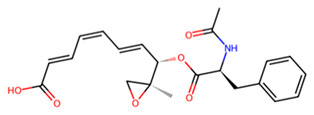	−7.6
	MMV979319	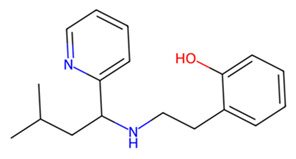	−7.2
L	UNPD73743	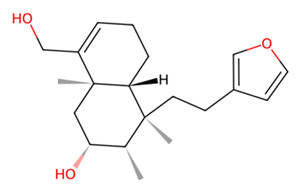	−7.4
	MMV1577465	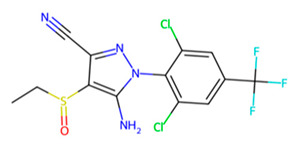	−6.8
B C L	E-64	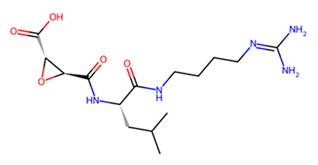	−6 −6.7 −5.3
D	Pepstatin	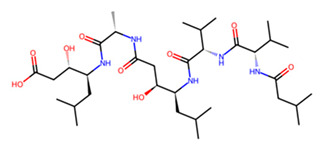	−7.2

## Data Availability

The datasets generated during the current study are available from the corresponding author upon reasonable request.

## Supplementary Materials

The following supporting information can be downloaded at https://www.mdpi.com/article/10.3390/pathogens13100850/s1, Figure S1: Predicted 3D structures of the designed MEVs targeting *Schistosoma* cercariae (a) and schistosomula (b) stage., Figure S2: An overlay of the binding poses of standard protease inhibitors E64 and pepstatin with *H. sapiens* cathepsins from X-ray structures and docking predictions.; Figure S3: 2D interaction of SmCC with hit compounds. (a) Interaction of E64 and UNPD28979 with SmCC. (b) Multiple sequence alignment of SmCC protein sequence against selected species., Figure S4: 2D interaction of SmCD with hit compounds. (a) Interaction of pepstatin, MMV979319 and UNPD125303 with SmCD. (b) Multiple sequence alignment of SmCD protein sequence against selected species., Figure S5: 2D interaction of SmCL with hit compounds. (a) Interaction of E64, MMV1577465 and UNPD73743 with SmCL. (b) Multiple sequence alignment of SmCL protein sequence against selected species., Table S1: Summary of Cercariae constructs Ramachandran plots statistics., Table S2: Summary of Schistosomula constructs Ramachandran plots statistics., Table S3: Epitopes selected for the construction of final vaccine candidates.; Supplementary datasheet S1: Highly expressed proteins in cercariae and schistosomula stages of *Schistosoma mansoni*.; Supplementary datasheet S2: Molecular docking results of compounds from MMV and SND libraries against *Schistosoma mansoni* cathepsins.
