# Supplementary material for: Computational Workflow to Design Novel Vaccine Candidates and Small-Molecule Therapeutics for Schistosomiasis
Source: Pathogens. 2024 Sep 30;13(10):850. doi: 10.3390/pathogens13100850 (PMC11509903; doi:10.3390/pathogens13100850)
Supplement: Supplementary file 1 [file pathogens-13-00850-s001.zip › supplementary_material.pdf]

Supplementary Material

Cercariae constructs

>Construct 1

APPHALS**EAAAK**HQHKRSIHSDFEQQPIMNTTITTSSS**KK**FANGYYSDYEQEKMTINST
YQSKPGIRSLYDVIMTSYFQMLGEFRLDDLAGDGSSCRDNGMCPQTSSRR**KK**DGSTT
SDSPQP**KK**EGPYEERSRTHVS**KK**KVINFDDASLKHTSNISAKNKHFISLDLAFLTFRIRT
E**KK**FSLYPKGPQLNYYQLTHQSQCLAQESRFEGVDCSIFSHP**KK**FLSKVFQITPLSLE**K**
KTTDNYMNGP**KK**QGIMYPKHTYVNLEAVKGIMLKPYFMLYGEVYAGEIDPIDWPIESQT
APFLEIV**KK**FSTLIQNTPTL**AAY**KITEAKIYV**AAY**IQDPTQIKI**AAY**SLIQDESHGI**AAY**ILIT
NLSEA**AAY**ATAKIIVKV**AAY**TLYLFIHVL**AAY**FLANISVYT**AAY**SLINNQYNL**AAY**MLWDFS
TYL**AAY**YLDNLIEQT**AAY**MLWDFSTYLA**AAY**SLWGSIYYC**AAY**SILDLIYFA**AAY**KLWES
KYLQLV**AAY**GLNKDFLLKV**AAY**KVADLQNQI**AAY**RVGPPVYFV**AAY**IMYPKHTYV**AAY**S
SFLSALKGNTLNGG**GPGPG**VNYYDKLTDELVNSN**GPGPG**GKDLRKCQAFKLPAT**GPG**
PGYKRFIVTDGSTTSDS**GPGPG**LKPYFMLYGEVYAGE

>Construct 2

APPHALS**EAAAK**HQHKRSIHSDFEQQPIMNTTITTSSS**KK**FANGYYSDYEQEKMTINST
YQSKPGIRSLYDVIMTSYFQMLGEFRLDDLAGDGSSCRDNGMCPQTSSRR**KK**DGSTT
SDSPQP**KK**EGPYEERSRTHVS**KK**KVINFDDASLKHTSNISAKNKHFISLDLAFLTFRIRT
E**KK**FSLYPKGPQLNYYQLTHQSQCLAQESRFEGVDCSIFSHP**KK**FLSKVFQITPLSLE**K**
KTTDNYMNGP**KK**QGIMYPKHTYVNLEAVKGIMLKPYFMLYGEVYAGEIDPIDWPIESQT
APFLEIV**KK**FSTLIQNTPTL**GPGPG**KITEAKIYV**GPGPG**IQDPTQIKI**GPGPG**SLIQDESH
GI**GPGPG**ILITNLSEA**GPGPG**ATAKIIVKV**GPGPG**TLYLFIHVL**GPGPG**FLANISVYT**GPG**
PGSLINNQYNL**GPGPG**MLWDFSTYL**GPGPG**YLDNLIEQT**GPGPG**MLWDFSTYLA**GPG**
PGSLWGSIYYC**GPGPG**SILDLIYFA**GPGPG**KLWESKYLQLV**GPGPG**GLNKDFLLKV**GP**
GPGKVADLQNQI**GPGPG**RVGPPVYFV**GPGPG**IMYPKHTYV**GPGPG**SSFLSALKGNTL
NGG**AAY**VNYYDKLTDELVNSN**AAY**GKDLRKCQAFKLPAT**AAY**YKRFIVTDGSTTSDS**A**
AYLKPYFMLYGEVYAGE

>Construct 3

APPHALS**EAAAK**HQHKRSIHSDFEQQPIMNTTITTSSS**AAY**FANGYYSDYEQEKMTINS
TYQSKPGIRSLYDVIMTSYFQMLGEFRLDDLAGDGSSCRDNGMCPQTSSRR**AAY**DGS
TTSDSPQP**AAY**EGPYEERSRTHVS**AAY**KVINFDDASLKHTSNISAKNKHFISLDLAFLTF
RIRTE**AAY**FSLYPKGPQLNYYQLTHQSQCLAQESRFEGVDCSIFSHP**AAY**FLSKVFQIT
PLSLE**AAY**TTDNYMNGP**AAY**QGIMYPKHTYVNLEAVKGIMLKPYFMLYGEVYAGEIDPI
DWPIESQTAPFLEIV**AAY**FSTLIQNTPTL**KK**KITEAKIYV**KK**IQDPTQIKI**KK**SLIQDESHGI
KKILITNLSEA**KK**ATAKIIVKV**KK**TLYLFIHVL**KK**FLANISVYT**KK**SLINNQYNL**KK**MLWDF
STYL**KK**YLDNLIEQT**KK**MLWDFSTYLA**KK**SLWGSIYYC**KK**SILDLIYFA**KK**KLWESKYLQ
LV**KK**GLNKDFLLKV**KK**KVADLQNQI**KK**RVGPPVYFV**KK**IMYPKHTYV**KK**SSFLSALKG
NTLNGG**GPGPG**VNYYDKLTDELVNSN**GPGPG**GKDLRKCQAFKLPAT**GPGPG**YKRFIV
TDGSTTSDS**GPGPG**LKPYFMLYGEVYAGE

>Construct 4

APPHALS**EAAAK**HQHKRSIHSDFEQQPIMNTTITTSSS**AAY**FANGYYSDYEQEKMTINS
TYQSKPGIRSLYDVIMTSYFQMLGEFRLDDLAGDGSSCRDNGMCPQTSSRR**AAY**DGS
TTSDSPQP**AAY**EGPYEERSRTHVS**AAY**KVINFDDASLKHTSNISAKNKHFISLDLAFLTF
RIRTE**AAY**FSLYPKGPQLNYYQLTHQSQCLAQESRFEGVDCSIFSHP**AAY**FSTLIQNTP
TL**GPGPG**KITEAKIYV**GPGPG**IQDPTQIKI**GPGPG**SLIQDESHGI**GPGPG**ILITNLSEA**GP**
GPGATAKIIVKV**GPGPG**TLYLFIHVL**GPGPG**FLANISVYT**GPGPG**SLINNQYNL**GPGPG**
MLWDFSTYL**GPGPG**YLDNLIEQT**GPGPG**MLWDFSTYLA**GPGPG**SLWGSIYYC**GPGPG**
SILDLIYFA**GPGPG**KLWESKYLQLV**GPGPG**GLNKDFLLKV**GPGPG**KVADLQNQI**GPGP**
GRVGPPVYFV**GPGPG**IMYPKHTYV**GPGPG**SSFLSALKGNTLNGG**KK**VNYYDKLTDEL
VNSN**KK**GKDLRKCQAFKLPAT**KK**YKRFIVTDGSTTSDS**KK**LKPYFMLYGEVYAGE

>Construct 5

APPHALS**EAAAK**HQHKRSIHSDFEQQPIMNTTITTSSS**GPGPG**FANGYYSDYEQEKMT
INSTYQSKPGIRSLYDVIMTSYFQMLGEFRLDDLAGDGSSCRDNGMCPQTSSRR**GPG**
PGDGSTTSDSPQP**GPGPG**EGPYEERSRTHVS**GPGPG**KVINFDDASLKHTSNISAKNK
HFISLDLAFLTFRIRTE**GPGPG**FSLYPKGPQLNYYQLTHQSQCLAQESRFEGVDCSIFS
HP**GPGPG**FLSKVFQITPLSLE**GPGPG**TTDNYMNGP**GPGPG**QGIMYPKHTYVNLEAVK
GIMLKPYFMLYGEVYAGEIDPIDWPIESQTAPFLEIV**GPGPG**FSTLIQNTPTL**AAY**KITEA
KIYV**AAY**IQDPTQIKI**AAY**SLIQDESHGI**AAY**ILITNLSEA**AAY**ATAKIIVKV**AAY**TLYLFIHVL
AAYFLANISVYT**AAY**SLINNQYNL**AAY**MLWDFSTYL**AAY**YLDNLIEQT**AAY**MLWDFSTYL
A**AAY**SLWGSIYYC**AAY**SILDLIYFA**AAY**KLWESKYLQLV**AAY**GLNKDFLLKV**AAY**KVADL
QNQI**AAY**RVGPPVYFV**AAY**IMYPKHTYV**AAY**SSFLSALKGNTLNGG**KK**VNYYDKLTDE
LVNSN**KK**GKDLRKCQAFKLPAT**KK**YKRFIVTDGSTTSDS**KK**LKPYFMLYGEVYAGE

> Construct 6

APPHALS**EAAAK**HQHKRSIHSDFEQQPIMNTTITTSSS**GPGPG**FANGYYSDYEQEKMT
INSTYQSKPGIRSLYDVIMTSYFQMLGEFRLDDLAGDGSSCRDNGMCPQTSSRR**GPG**
PGDGSTTSDSPQP**GPGPG**EGPYEERSRTHVS**GPGPG**KVINFDDASLKHTSNISAKNK
HFISLDLAFLTFRIRTE**GPGPG**FSLYPKGPQLNYYQLTHQSQCLAQESRFEGVDCSIFS
HP**GPGPG**FLSKVFQITPLSLE**GPGPG**TTDNYMNGP**GPGPG**QGIMYPKHTYVNLEAVK
GIMLKPYFMLYGEVYAGEIDPIDWPIESQTAPFLEIV**GPGPG**FSTLIQNTPTL**KK**KITEAKI
YV**KK**IQDPTQIKI**KK**SLIQDESHGI**KK**ILITNLSEA**KK**ATAKIIVKV**KK**TLYLFIHVL**KK**FLAN
ISVYT**KK**SLINNQYNL**KK**MLWDFSTYL**KK**YLDNLIEQT**KK**MLWDFSTYLA**KK**SLWGSIY
YC**KK**SILDLIYFA**KK**KLWESKYLQLV**KK**GLNKDFLLKV**KK**KVADLQNQI**KK**RVGPPVYF
V**KK**IMYPKHTYV**KK**SSFLSALKGNTLNGG**AAY**VNYYDKLTDELVNSN**AAY**GKDLRKCQ
AFKLPAT**AAY**YKRFIVTDGSTTSDS**AAY**LKPYFMLYGEVYAGE

Schistosomula constructs

>Construct 1

APPHALS**EAAAK**SRENDQYKN**KK**HNNLSTDHYNNST**KK**GLLNSKNTDKKMDVTR**KK**
PFDFDDDKTESNDEYDGHTYDDLLLPIPCCMMDKHFRLIDINCPNFHYMSENEQKHHN
KGCKQ**KK**LKYYYEKNEYGD**KK**DQYASDVQQS**KK**SGAAAGIAVGA**KK**SGSLFLTEALK
WNTW**KK**TIRTYVQDKLQVTSRSCVPVCVEADARRSGSGIVTSC**KK**LLCPNPFNPSSP
LVHNYSN**KK**TLYLFIHVL**AAY**FLANISVYT**AAY**VLNPPKPGL**AAY**FAASVLLKV**AAY**VISS
LPVNL**AAY**YLKFVNITV**AAY**TMYVAVWAV**AAY**TLWPDHYKPIV**AAY**VLDMYIWHL**AAY**Y
LSDYKIRTYV**AAY**MVYDSHVAL**AAY**YLTQQFYMA**AAY**YLSDYKIRT**AAY**SLLKFTTLV**AA**
YSLIYDSLILL**AAY**LIYDSLILL**AAY**FSLIYDSLILL**AAY**FMDKYPDATV**AAY**ILYYFQPEL**AA**
YFLYTFNAFV**AAY**RLLSQEHDNNL**AAY**SLFAAVVMI**AAY**HLYSFTFQI**AAY**KLEETAFGI**A**
AYSYLTEGNIFL**AAY**GSYLTEGNIFL**AAY**LLIPYSCRV**AAY**MLTVIVVTL**AAY**LLMTIITPI**A**
AYRRVYVSGVPENAPPG**GPGPG**TEPFQTIWPSDLPKQ

>Construct 2

APPHALS**EAAAK**SRENDQYKN**KK**HNNLSTDHYNNST**KK**GLLNSKNTDKKMDVTR**KK**
PFDFDDDKTESNDEYDGHTYDDLLLPIPCCMMDKHFRLIDINCPNFHYMSENEQKHHN
KGCKQ**KK**LKYYYEKNEYGD**KK**DQYASDVQQS**KK**SGAAAGIAVGA**KK**SGSLFLTEALK
WNTW**KK**TIRTYVQDKLQVTSRSCVPVCVEADARRSGSGIVTSC**KK**LLCPNPFNPSSP
LVHNYSN**KK**TLYLFIHVL**GPGPG**FLANISVYT**GPGPG**VLNPPKPGL**GPGPG**FAASVLLK
V**GPGPG**VISSLPVNL**GPGPG**YLKFVNITV**GPGPG**TMYVAVWAV**GPGPG**TLWPDHYKPI
V**GPGPG**VLDMYIWHL**GPGPG**YLSDYKIRTYV**GPGPG**MVYDSHVAL**GPGPG**YLTQQFY
MA**GPGPG**YLSDYKIRT**GPGPG**SLLKFTTLV**GPGPG**SLIYDSLILL**GPGPG**LIYDSLILL**GP**
GPGFSLIYDSLILL**GPGPG**FMDKYPDATV**GPGPG**ILYYFQPEL**GPGPG**FLYTFNAFV**GP**
GPGRLLSQEHDNNL**GPGPG**SLFAAVVMI**GPGPG**HLYSFTFQI**GPGPG**KLEETAFGI**GP**
GPGSYLTEGNIFL**GPGPG**GSYLTEGNIFL**GPGPG**LLIPYSCRV**GPGPG**MLTVIVVTL**GP**
GPGLLMTIITPI**GPGPG**RRVYVSGVPENAPPG**AAY**TEPFQTIWPSDLPKQ

>Construct 3

APPHALS**EAAAK**SRENDQYKN**AAY**HNNLSTDHYNNST**AAY**GLLNSKNTDKKMDVTR**A**
AYPFDFDDDKTESNDEYDGHTYDDLLLPIPCCMMDKHFRLIDINCPNFHYMSENEQKH
HNKGCKQ**AAY**LKYYYEKNEYGD**AAY**DQYASDVQQS**AAY**SGAAAGIAVGA**AAY**SGSLF
LTEALKWNTW**AAY**TIRTYVQDKLQVTSRSCVPVCVEADARRSGSGIVTSC**AAY**LLCPN
PFNPSSPLVHNYSN**AAY**TLYLFIHVL**KK**FLANISVYT**KK**VLNPPKPGL**KK**FAASVLLKV**K**
KVISSLPVNL**KK**YLKFVNITV**KK**TMYVAVWAV**KK**TLWPDHYKPIV**KK**VLDMYIWHL**KK**Y
LSDYKIRTYV**KK**MVYDSHVAL**KK**YLTQQFYMA**KK**YLSDYKIRT**KK**SLLKFTTLV**KK**SLIY
DSLILL**KK**LIYDSLILL**KK**FSLIYDSLILL**KK**FMDKYPDATV**KK**ILYYFQPEL**KK**FLYTFNAF
V**KK**RLLSQEHDNNL**KK**SLFAAVVMI**KK**HLYSFTFQI**KK**KLEETAFGI**KK**SYLTEGNIFL**K**
KGSYLTEGNIFL**KK**LLIPYSCRV**KK**MLTVIVVTL**KK**LLMTIITPI**KK**RRVYVSGVPENAPP
G**GPGPG**TEPFQTIWPSDLPKQ

>Construct 4

APPHALS**EAAAK**SRENDQYKN**AAY**HNNLSTDHYNNST**AAY**GLLNSKNTDKKMDVTR**A**
AYPFDFDDDKTESNDEYDGHTYDDLLLPIPCCMMDKHFRLIDINCPNFHYMSENEQKH
HNKGCKQ**AAY**LKYYYEKNEYGD**AAY**DQYASDVQQS**AAY**SGAAAGIAVGA**AAY**SGSLF

LTEALKWNTW**AAY**TIRTYVQDKLQVTSRSCVPVCVEADARRSGSGIVTSC**AAY**LLCPN
PFNPSSPLVHNYSN**AAY**TLYLFIHVL**GPGPG**FLANISVYT**GPGPG**VLNPPKPGL**GPGPG**
FAASVLLKV**GPGPG**VISSLPVNL**GPGPG**YLKFVNITV**GPGPG**TMYVAVWAV**GPGPG**TL
WPDHYKPIV**GPGPG**VLDMYIWHL**GPGPG**YLSDYKIRTYV**GPGPG**MVYDSHVAL**GPGP**
GYLTQQFYMA**GPGPG**YLSDYKIRT**GPGPG**SLLKFTTLV**GPGPG**SLIYDSLILL**GPGPG**LI
YDSLILL**GPGPG**FSLIYDSLILL**GPGPG**FMDKYPDATV**GPGPG**ILYYFQPEL**GPGPG**FLY
TFNAFV**GPGPG**RLLSQEHDNNL**GPGPG**SLFAAVVMI**GPGPG**HLYSFTFQI**GPGPG**KLE
ETAFGI**GPGPG**SYLTEGNIFL**GPGPG**GSYLTEGNIFL**GPGPG**LLIPYSCRV**GPGPG**MLT
VIVVTL**GPGPG**LLMTIITPI**GPGPG**RRVYVSGVPENAPPG**KK**TEPFQTIWPSDLPKQ

>Construct 5

APPHALS**EAAAK**SRENDQYKN**GPGPG**HNNLSTDHYNNST**GPGPG**GLLNSKNTDKKM
DVTR**GPGPG**PFDFDDDKTESNDEYDGHTYDDLLLPIPCCMMDKHFRLIDINCPNFHYM
SENEQKHHNKGCKQ**GPGPG**LKYYYEKNEYGD**GPGPG**DQYASDVQQS**GPGPG**SGAA
AGIAVGA**GPGPG**SGSLFLTEALKWNTW**GPGPG**TIRTYVQDKLQVTSRSCVPVCVEAD
ARRSGSGIVTSC**GPGPG**LLCPNPFNPSSPLVHNYSN**GPGPG**TLYLFIHVL**AAY**FLANIS
VYT**AAY**VLNPPKPGL**AAY**FAASVLLKV**AAY**VISSLPVNL**AAY**YLKFVNITV**AAY**TMYVAV
WAV**AAY**TLWPDHYKPIV**AAY**VLDMYIWHL**AAY**YLSDYKIRTYV**AAY**MVYDSHVAL**AAY**Y
LTQQFYMA**AAY**YLSDYKIRT**AAY**SLLKFTTLV**AAY**SLIYDSLILL**AAY**LIYDSLILL**AAY**FSLI
YDSLILL**AAY**FMDKYPDATV**AAY**ILYYFQPEL**AAY**FLYTFNAFV**AAY**RLLSQEHDNNL**AA**
YSLFAAVVMI**AAY**HLYSFTFQI**AAY**KLEETAFGI**AAY**SYLTEGNIFL**AAY**GSYLTEGNIFL**A**
AYLLIPYSCRV**AAY**MLTVIVVTL**AAY**LLMTIITPI**AAY**RRVYVSGVPENAPPG**KK**TEPFQTI
WPSDLPKQ

>Construct 6

APPHALS**EAAAK**SRENDQYKN**GPGPG**HNNLSTDHYNNST**GPGPG**GLLNSKNTDKKM
DVTR**GPGPG**PFDFDDDKTESNDEYDGHTYDDLLLPIPCCMMDKHFRLIDINCPNFHYM
SENEQKHHNKGCKQ**GPGPG**LKYYYEKNEYGD**GPGPG**DQYASDVQQS**GPGPG**SGAA
AGIAVGA**GPGPG**SGSLFLTEALKWNTW**GPGPG**TIRTYVQDKLQVTSRSCVPVCVEAD
ARRSGSGIVTSC**GPGPG**LLCPNPFNPSSPLVHNYSN**GPGPG**TLYLFIHVL**KK**FLANISV
YT**KK**VLNPPKPGL**KK**FAASVLLKV**KK**VISSLPVNL**KK**YLKFVNITV**KK**TMYVAVWAV**KK**
TLWPDHYKPIV**KK**VLDMYIWHL**KK**YLSDYKIRTYV**KK**MVYDSHVAL**KK**YLTQQFYMA**K**
KYLSDYKIRT**KK**SLLKFTTLV**KK**SLIYDSLILL**KK**LIYDSLILL**KK**FSLIYDSLILL**KK**FMDKY
PDATV**KK**ILYYFQPEL**KK**FLYTFNAFV**KK**RLLSQEHDNNL**KK**SLFAAVVMI**KK**HLYSFTF
QI**KK**KLEETAFGI**KK**SYLTEGNIFL**KK**GSYLTEGNIFL**KK**LLIPYSCRV**KK**MLTVIVVTL**KK**
LLMTIITPI**KK**RRVYVSGVPENAPPG**AAY**TEPFQTIWPSDLPKQ

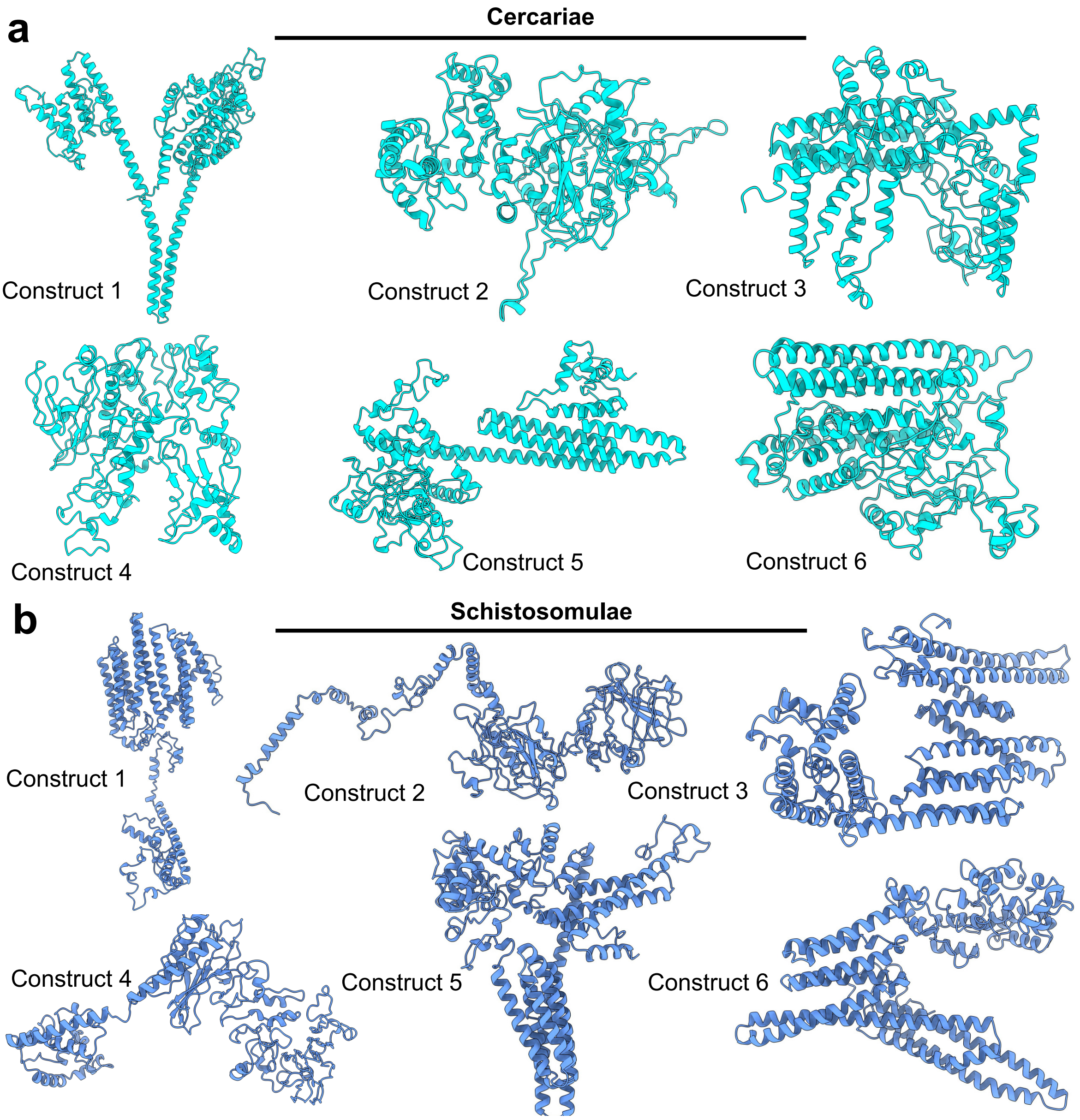

Figure S1: Predicted 3D structures of the designed MEVs targeting Schistosoma cercariae **(a)** and schistosomulae **(b)** stage.

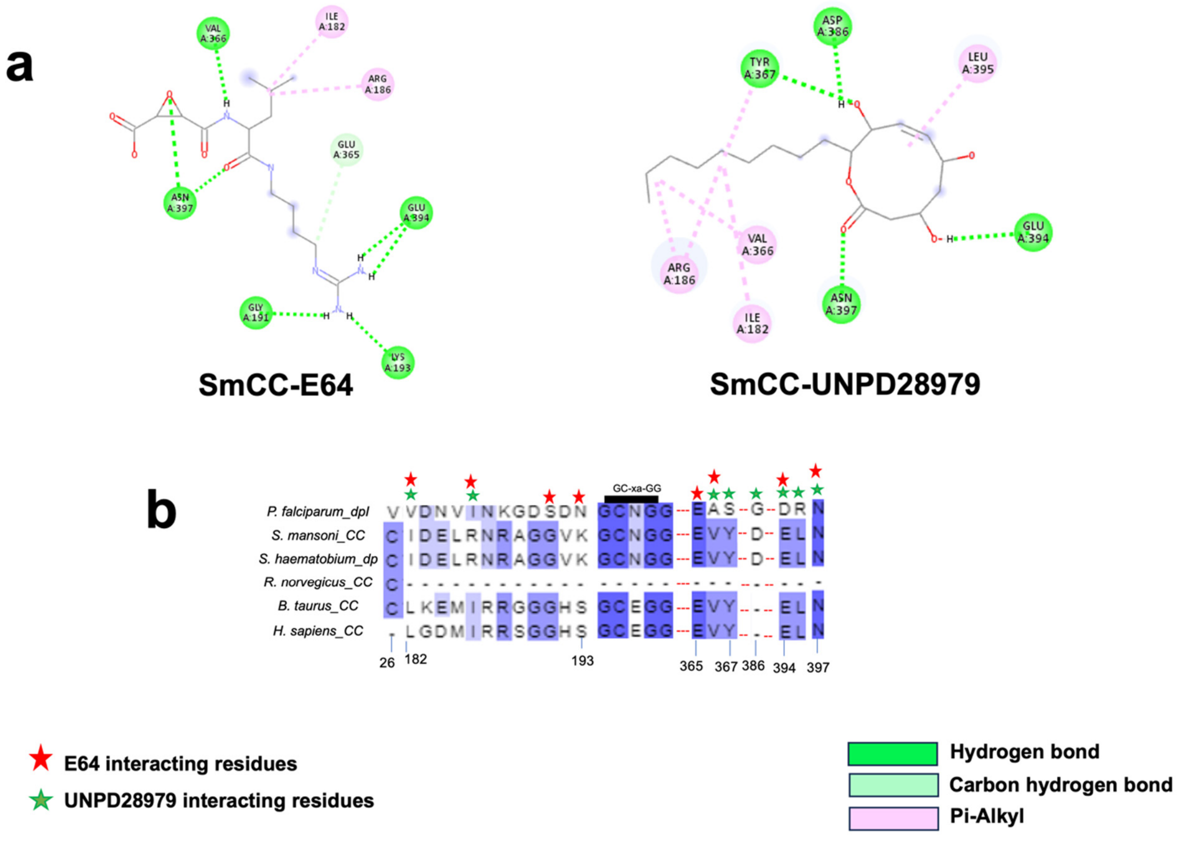

Figure S2: 2D interaction of SmCC with hit compounds**. (a)** Interaction of E64 and UNPD28979 with SmCC**. (b)** Multiple sequence alignment of SmCC protein sequence against selected species.

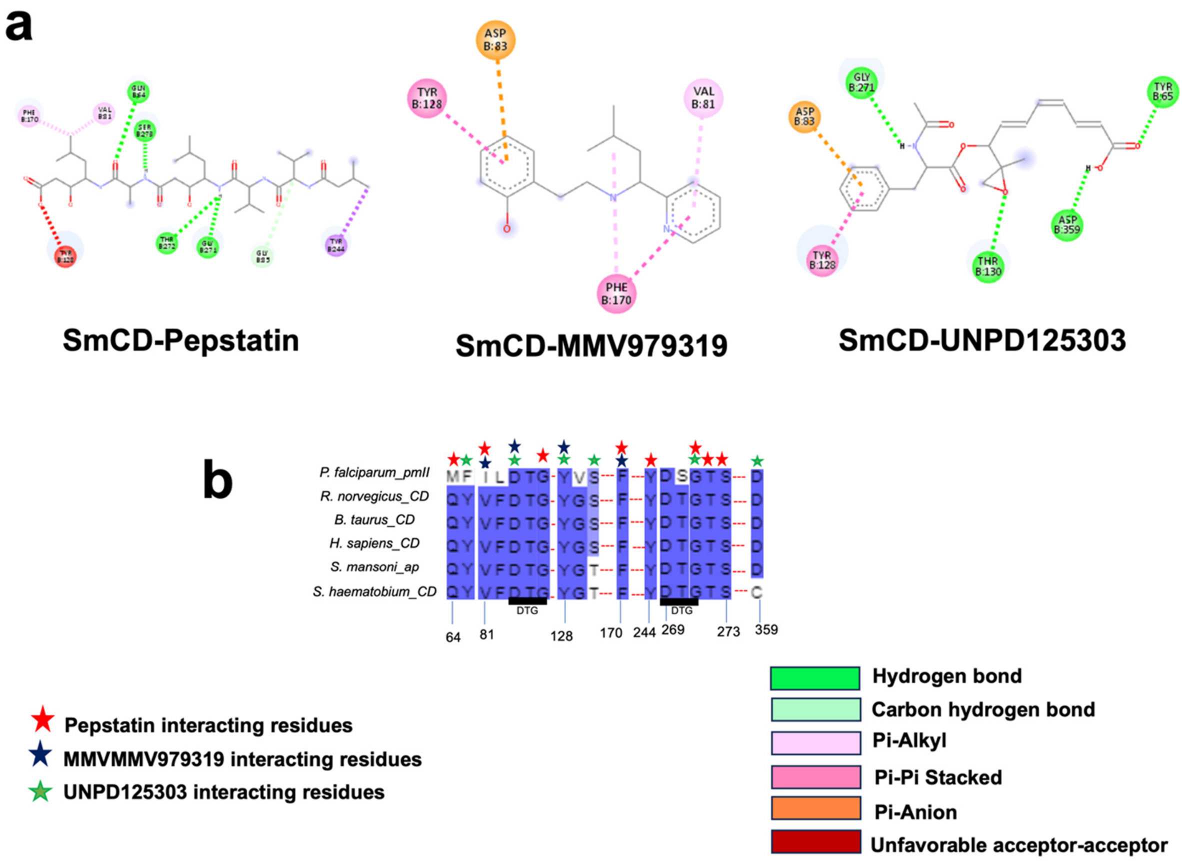

Figure S3: 2D interaction of SmCD with hit compounds**. (a)** Interaction of pepstatin, MMV979319 and UNPD125303 with SmCD**. (b)** Multiple sequence alignment of SmCD protein sequence against selected species.

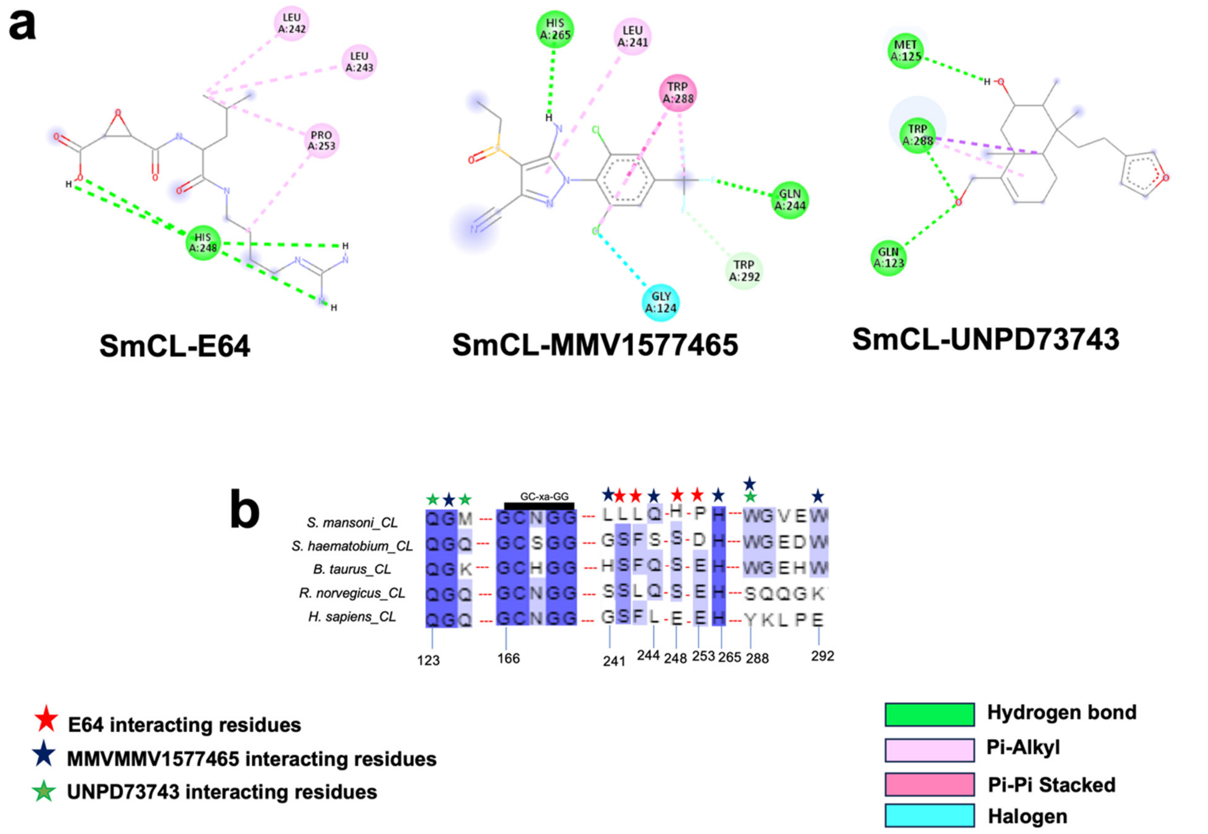

Figure S4: 2D interaction of SmCL with hit compounds**. (a)** Interaction of E64, MMV1577465 and UNPD73743 with SmCL**. (b)** Multiple sequence alignment of SmCL protein sequence against selected species.

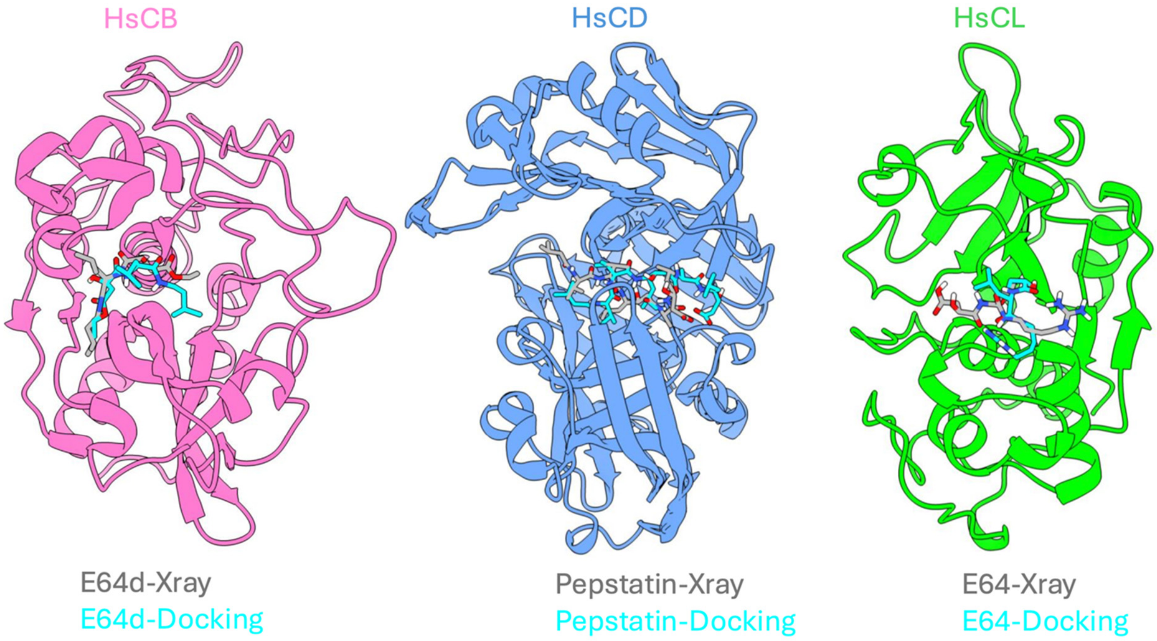

Figure S5: An overlay of the binding poses of standard protease inhibitors E64 and pepstatin with *H. sapien* cathepsins from X-ray structures and docking predictions. The PDB codes for HsCB, HsCD, and HsCL are 8HEI, 1LYB, and 8A4V, respectively.

Table S1: Epitopes selected for the construction of final vaccine candidates

***Schistosoma mansoni* stage**	**Linear B-cell epitopes**	**Cytotoxic T-lymphocyte epitopes**	**Helper T-lymphocyte epitopes**
**Cercariae**	1. HQHKRSIHSDFEQQPIMNTTITTSSS<br>2. FANGYYSDYEQEKMTINSTYQSKPGIRSLYDVIMTSYFQMLGEFRLDDLAGDGSSCRDNGMCPQTSSRR<br>3. DGSTTSDSPQP<br>4. EGPYEERSRTHVSK<br>5. VINFDDASLKHTSNISAKNKHFISLDLAFLTFRIRTE<br>6. FSLYPKGPQLNYYQLTHQSQCLAQESRFEGVDCSIFSHP<br>7. FLSKVFQITPLSLE<br>8. TTDNYMNGP<br>9. QGIMYPKHTYVNLEAVKGIMLKPYFMLYGEVYAGEIDPIDWPIESQTAPFLEIV	1. FSTLIQNTPTL<br>2. KITEAKIYV<br>3. IQDPTQIKI<br>4. SLIQDESHGI<br>5. ILITNLSEA<br>6. ATAKIIVKV<br>7. TLYLFIHVL<br>8. FLANISVYT<br>9. SLINNQYNL<br>10. MLWDFSTYL<br>11. YLDNLIEQT<br>12. MLWDFSTYLA<br>13. SLWGSIYYC<br>14. SILDLIYFA<br>15. KLWESKYLQLV<br>16. GLNKDFLLKV<br>17. KVADLQNQI<br>18. RVGPPVYFV<br>19. IMYPKHTYV	1. SSFLSALKGNTLNGG<br>2. VNYYDKLTDELVNSN<br>3. GKDLRKCQAFKLPAT<br>4. YKRFIVTDGSTTSDS<br>5. LKPYFMLYGEVYAGE
**Schistosomula**	1. SRENDQYKN<br>2. HNNLSTDHYNNST<br>3. GLLNSKNTDKKMDVTR<br>4. PFDFDDDKTESNDEYDGHTYDDLLLPIPCCMMDKHFRLIDINCPNFHYMSENEQKHHNKGCKQ<br>5. LKYYYEKNEYGD<br>6. DQYASDVQQS<br>7. SGAAAGIAVGA<br>8. SGSLFLTEALKWNTW<br>9. TIRTYVQDKLQVTSRSCVPVCVEADARRSGSGIVTSC<br>10. LLCPNPFNPSSPLVHNYSN	1. TLYLFIHVL<br>2. FLANISVYT<br>3. VLNPPKPGL<br>4. FAASVLLKV<br>5. VISSLPVNL<br>6. YLKFVNITV<br>7. TMYVAVWAV<br>8. TLWPDHYKPIV<br>9. VLDMYIWHL<br>10. YLSDYKIRTYV<br>11. MVYDSHVAL<br>12. YLTQQFYMA<br>13. YLSDYKIRT<br>14. SLLKFTTLV<br>15. SLIYDSLILL<br>16. LIYDSLILL<br>17. FSLIYDSLILL<br>18. FMDKYPDATV<br>19. ILYYFQPEL	1. RRVYVSGVPENAPPG<br>2. TEPFQTIWPSDLPKQ

20. FLYTFNAFV
21. RLLSQEHDNNL
22. SLFAAVVMI
23. HLYSFTFQI
24. KLEETAFGI
25. SYLTEGNIFL
26. GSYLTEGNIFL
27. LLIPYSCRV
28. MLTVIVVTL
29. LLMTIITPI

Table S2: Summary of Cercariae constructs Ramachandran plots statistics

Multi-epitope antigen (Constructs)	No. of residues in the favored region	No. of residues in the allowed region	No. of residues in the disallowed region	Epitopes composition/linkers
1	496 (88.6%)	62 (11.1%)	2 (0.4%)	9 LBL/**KK**<br>19 CTL/**AAY**<br>5 HTL (1 SB and 4 WB)/**GPGPG**
2	416 (80.8%)	95 (18.5%)	4 (0.8%)	9 LBL/**KK**<br>19 CTL/**GPGPG**<br>5 HTL (1 SB and 4 WB)/**AAY**
3	489 (88.9%)	58 (10.5%)	3 (0.5%)	9 LBL/**AAY**<br>19 CTL/**KK**<br>5 HTL (1 SB and 4 WB)/**GPGPG**
4	355 (79.6%)	85 (19.0%)	6 (1.3%)	9LBL/**AAY**<br>19CTL/**GPGPG**<br>5 HTL (1 SB and 4 WB)/**KK**
5	461 (83.8%)	86 (15.6%)	3 (0.5%)	9 LBL/**GPGPG**<br>19 CTL/**AAY**<br>5 HTL (1 SB and 4 WB)/**KK**
6	457 (85.4%)	76 (14.2%)	2 (0.4%)	9 LBL/**GPGPG**<br>19 CTL/**KK**<br>5 HTL (1 SB and 4 WB)/**AAY**

Keys

- LBL-linear B-lymphocyte epitopes
- CTL-Cytotoxic T-lymphocyte epitopes
- HTL- Helper T-lymphocyte epitopes

Table S3: Summary of Schistosomula constructs Ramachandran plots statistics

Multi-epitope antigen	No. of residues in the favored region	No. of residues in the allowed region	No. of residues in the disallowed region	Epitopes composition/linkers
Construct 1	510 (87.6%)	69 (11.9%)	3 (0.5%)	10 LBL/**KK**<br>29 CTL/**AAY**<br>2 HTL (1 SB, 1 WB)/**GPGPG**
Construct 2	364 (73.1%)	128 (25.7%)	6 (1.2%)	10 LBL/**KK**<br>29 CTL/**GPGPG**<br>2 HTL (1 SB, 1 WB)/**AAY**
Construct 3	495 (87.9%)	65 (11.5%)	3 (0.5%)	10 LBL/**AAY**<br>29 CTL/**KK**<br>2 HTL (1 SB, 1 WB)/**GPGPG**
Construct 4	399 (78.7%)	106 (20.9%)	2 (0.4%)	10 LBL/**AAY**<br>29 CTL/**GPGPG**<br>2 HTL (1 SB, 1 WB)/**KK**
Construct 5	505 (89.5%)	54 (9.6%)	5 (0.9%)	10 LBL/**GPGPG**<br>29 CTL/**AAY**<br>2 HTL (1 SB, 1 WB)/**KK**
Construct 6	490 (91.4%)	42 (7.8%)	4 (0.7%)	10 LBL/**GPGPG**<br>29 CTL/**KK**<br>2 HTL (1 SB, 1 WB)/**AAY**

Keys

- LBL-linear B-lymphocyte epitopes
- CTL-Cytotoxic T-lymphocyte epitopes
- HTL- Helper T-lymphocyte epitoes
